# Physical activity promotion in rural health care settings: A rapid realist review

**DOI:** 10.1016/j.pmedr.2022.101905

**Published:** 2022-07-09

**Authors:** Chelsea Pelletier, Katie Cornish, Tess Amyot, Anne Pousette, Gloria Fox, David Snadden, Taru Manyanga

**Affiliations:** a School of Health Sciences, University of Northern British Columbia, Prince George, Canada; b Faculty of Medicine, University of British Columbia, Vancouver, Canada; c Health Research Institute, University of Northern British Columbia, Prince George, Canada; d Promotion of Wellness in Northern BC, Prince George, Canada; e Population and Preventive Public Health, Northern Health, Prince George, Canada; f Division of Medical Sciences, University of Northern British Columbia, Prince George, Canada

**Keywords:** Physical activity, Rural populations, Knowledge synthesis, Exercise is medicine

## Abstract

Physical activity promotion in health care settings is poorly understood and has limited uptake among health care providers. The environmental and health care context of rural communities is unique from urban areas and may interact to influence intervention delivery and success. The aim of this rapid realist review was to synthesize knowledge related to the promotion of physical activity in rural health and social care settings. We searched Medline EBSCO, CINAHL, PsychINFO, and SPORTDiscus for relevant publications. We included qualitative or quantitative studies reporting on an intervention to promote physical activity in rural health (e.g., primary or community care) or social (e.g., elder support services) care settings. Studies without a rural focus or well-defined physical activity/exercise component were excluded. Populations of interest included adults and children in the general population or clinical sub-population. Intervention mechanisms from included studies were mapped to the Behaviour Change Wheel (capability, opportunity, motivation (COM-B)). Twenty studies were included in our review. Most interventions focused on older adults or people with chronic disease risk factors. The most successful intervention strategies leading to increased physical activity behaviour included wearable activity trackers, and check-ins or reminders from trusted sources. Interventions with mechanisms categorized as physical opportunity, automatic motivation, and psychological capability were more likely to be successful than other factors of the COM-B model. Successful intervention activities included a method for tracking progress, providing counselling, and follow-up reminders to prompt behaviour change. Cultivation of necessary community partnerships and adaptations for implementation of interventions in rural communities were not clearly described and may support successful outcomes in future studies.

## Highlights

• Rural communities have a unique health care and physical activity context.
• We conducted a rapid realist review in partnership with knowledge users.
• Check-ins from health care providers may lead to intervention success and are valued by participants.
• A method for tracking progress is an important component of interventions delivered in rural health care settings.

## 1 Introduction

Physical activity is one of the most important strategies for preventing and managing noncommunicable disease and reducing all-cause mortality (Chastin et al., 2021, Lee et al., 2012). Despite decades of research, advocacy, and guideline development, physical inactivity remains a considerable global health challenge (Kohl et al., 2012). Examination of physical activity participation reveals inequities driven by personal circumstances, environmental context, and policy factors shaping physical activity opportunities for individuals and groups (Sfm et al., 2020). Rural residents have fewer opportunities for physical activity participation due to reduced access to indoor facilities and limited active transportation infrastructure (Pelletier et al., 2021), a lower odds of meeting physical activity guidelines (Pelletier et al., 2021), and a lower overall health status when compared to their urban counterparts (Pong et al., 2009). The development and adaptation of evidence-based interventions for physical activity promotion in rural communities is an essential strategy to address global physical activity inequities and remains a substantial knowledge gap (Pelletier et al., 2020).

### 1.1 Physical activity promotion in health care settings

Health care providers are key messengers and advocates of health-enhancing opportunities (Vuori et al., 2013). Advice from respected health care professionals can positively impact physical activity participation in patients, especially when it includes multiple behaviour change components such as written exercise prescriptions and counselling (Sanchez et al., 2015). While physical activity promotion in health care settings is one of the eight investments that work to promote physical activity identified by the International Society for Physical Activity and Health (Milton et al., 2021), many reviews conclude limited effectiveness of counselling in primary care settings (van der Wardt et al., 2021), and uptake among primary care providers is low (Lobelo et al., 2018). Barriers to integrating physical activity promotion into primary and community care settings include lack of training and the usability and/or fit of currently available tools with local practice (Lion et al., 2019). To be successful, interventions delivered in primary and community care settings must be tailored to the physical activity experiences and circumstances of community members, and the role and scope of practice of health care providers considering the sociopolitical and organizational culture, resources, and support available (Moreno-Peral et al., 2015, Huijg et al., 2015).

### 1.2 Rural health care context

The concept of rurality has developed from a simple measure of ‘non-urban’ to include various quantifiable characteristics (e.g., population density, infrastructure, distance to regional centres) (Nelson et al., 2021, Gessert et al., 2015). Although quantitative measures are more easily operationalized for research purposes, these definitions fail to incorporate the heterogeneity of rural communities and the varied social and cultural aspects influencing community structure (Nelson et al., 2021, Gessert et al., 2015). Definitions of rurality in the context of health care have varied meaning with reference to technology available, density of health care providers to patients, accessibility of information, and access to specialists (Hart et al., 2005, Chen et al., 2019). Successful health care delivery in rural communities is characterized by patient and provider autonomy, avoidance of a top-down or paternalistic approach, and respectful relationships – incorporating aspects of social care and reflecting a holistic approach to health (Johnston et al., 2021).

### 1.3 Physical activity promotion in rural communities

Understanding how health care and physical activity contexts in rural communities interact to influence the success of interventions will help advance effectiveness and address persistent health inequities in rural communities. While there are numerous recent reviews on physical activity promotion in primary care by physicians (Sanchez et al., 2015) or other health care providers (Crisford et al., 2018), these reviews rarely explore how community context impacts intervention delivery (e.g., reflect on how the intervention may be applicable to communities of different sizes or location), identify intervention mechanisms, or provide a nuanced focus on rural health care settings.

### 1.4 Purpose

The purpose of this review was to synthesize knowledge related to the promotion of physical activity in rural health and social care settings by exploring what works, for whom, and in what circumstances. Our review was guided by the following research questions:

1. What interventions work to promote physical activity in rural health and social care settings?
2. What are the mechanisms and contextual factors impacting the delivery of physical activity interventions in rural health and social care settings?

## 2 Methods

The purpose of a rapid realist review is to determine not only what makes an intervention successful, but also the mechanisms, contexts, key attributes, and contributing factors promoting a successful outcome (Pawson and Manzano-Santaella, 2012, Pawson et al., 2005). Compared to a traditional realist review, a rapid realist review is designed to be conducted in a shorter time frame and balance comprehensiveness and speed. To support contextualization of findings, the rapid realist approach is strengthened by engagement of knowledge users to understand how and why interventions work in a specific context (Saul et al., 2013). The methodology of rapid realist reviews acknowledges the inherent complexities of interventions by considering ‘what works, for whom, in what contexts, to what extent, and most importantly how and why’ by considering different types of evidence (Pawson et al., 2005). A realist review was appropriate for this project given the complexity of physical activity behaviour and the unique social-cultural environment of rural communities shaping health care and physical activity experiences. A rapid form of realist review was chosen due to the increased focuse on community partnerships to inform context, and to align with funding timeframes and knowledge user needs.

Our rapid realist review followed the ten steps described by Saul and colleagues (Saul et al., 2013) which included: developing scope and research questions, identifying how findings and recommendations will be used, developing search terms, identifying articles and documents for inclusion, quality review, extracting data, validation of findings, synthesis, and dissemination. We followed the PRISMA reporting items for systematic reviews (Page et al., 2021) and RAMESES guidelines for reporting realist syntheses (Wong et al., 2013). This review was prospectively registered with PROSPERO (CRD42021240987).

### 2.1 Knowledge user involvement

A key component of a rapid realist review is the engagement of relevant partners and knowledge users in the review process (Saul et al., 2013). Our collaborative review team included the research team (composed of researchers and clinician scientists), an advisory group of rural clinicians and health care practitioners, and the Knowledge Synthesis Centre at the University of Northern British Columbia Health Research Institute following an approach we have used previously (Cornish et al., 2020). The advisory group provided feedback on project aims, definitions, inclusion/exclusion criteria, assisted in interpretation and contextualization of findings, and provided input on key issues as the review progressed. To allow flexibility for each advisory group member and accommodate dynamic time demands, we provided options for engagement including virtual meetings, asynchronous document review, and brief summaries identifying important target areas with guiding questions.

### 2.2 Search and study selection

Using keywords and subject headings for pre-identified population (e.g., primary care provider), concept (e.g., physical activity counselling), and context (e.g., rural), we searched Medline EBSCO, CINAHL, PsychINFO, and SPORTDiscus for publications relevant to our review on May 4, 2021 (see supplementary file 1 for Medline search strategy). We hand searched reference lists from key publications identified by the advisory group, the research team, and included papers. Publications were uploaded into DistillerSR (Evidence Partners, Ottawa) where they underwent two levels of screening: 1) title and abstract, and 2) full-text. Screening was based on the definitions adopted for this review (Table 1), and inclusion and exclusion criteria (Table 2). We decided to include articles describing a study occurring in traditional health care settings (e.g., medical clinic) and in social or community care (e.g., supports for activities of daily living). We intentionally considered health care systems and services beyond a traditional biomedical approach to be inclusive and respectful of different meanings of health and health care delivery. Our research team and advisory group decided a holistic approach to health was a relevant lens when considering interventions delivered in rural areas where providers may work outside of a traditional scope of practice to consider concepts such as social wellbeing. Our inclusion of populations across the lifespan was also intentional as the resources, population composition, and environment differ from community to community, and we wanted to capture interventions applicable to all community members .

**Table 1:** Definitions of key terms and concepts adopted for this review.

Concept	Definition
Rural	We consider rurality as a concept beyond population size. We acknowledge rurality is conceptualized for different regions and communities based on relationships, culture, and identity. For the purposes of this review, we will include any paper taking place in a rural or remote community as identified by the study authors.
Health care setting	We take a broad view of health care settings, recognizing different models of health care delivery across countries, regions, and within a rural setting. We consider a primary or community care centre as a location providing health services by physicians, nurses, and other health care providers in private or public settings. We recognize team-based approaches to care delivery spanning outside formal health care settings into the community.
Social care setting	We define social care as services related to long-term inpatient care, programming for older adults, and supports for people with chronic disease or disability to aid with activities of daily living and/or providing other support services. Social services may include or not include a specific health-care component, considered broadly within the specific regional and national context and regulations as defined by each study.
Health care provider	All workers engaged in delivery of health or social care services working in individual or team environment and within formal primary, community, or social care settings. We consider the term health care provider (or health care worker/professional) broadly and within the specific regional and national context and regulations as defined by each study.

**Table 2:** Inclusion and exclusion criteria.

Inclusion	Exclusion
1. Studies involving the promotion of physical activity by rural health or social care providers in a health care or community setting to the general population or a clinical sub-group will be included. Studies must: a. identify an intervention, program, or approach implemented to promote physical activity b. be promoted or initiated by health care providers (individual or interdisciplinary team approach) c. take place in a rural, remote, northern or Indigenous health care setting or community d. be undertaken with general population or any clinical sub-group (no age, gender, or risk factor parameters) e. describe outcomes of interest – change in physical activity behaviour, health outcomes, or implementation outcomes (e.g., feasibility, user experience) f. interventions/programs/approaches may include behaviour change interventions, web or telehealth (virtual delivery), counselling, referrals, educational interventions or physical activity prescriptions g. multi-component interventions are eligible provided the physical activity/exercise component of the intervention is well described and outcomes reported separately 2. Peer reviewed academic publications; all methods (quantitative, qualitative, and mixed methods) 3. Studies written in English	1. Studies conducted in urban or metropolitan settings or studies including both urban and rural communities/areas, but no strategy developed or adapted to rural, remote, northern, or Indigenous setting 2. Studies, programs, or interventions promoted by other groups (volunteer, community organizations, etc.) 3. Studies focused on general lifestyle interventions without a specific focus on physical activity, or where physical activity component of intervention not well designed or evaluated (e.g., no physical activity outcomes) 4. Case reports, conference abstracts, editorial and opinion pieces, literature reviews, book chapters, book reviews, and book synopses will be excluded 5. Non-English studies 6. Secondary exclusion – unable to locate full text

We pilot tested our screening process and criteria with 10% of the sample to confirm clarity and test reviewer compatibility. Screening was completed by two independent reviewers and disagreements were resolved by a third reviewer. The Kappa score at level one indicated moderate agreement (0.59), and the Kappa score for level two indicated almost perfect agreement (0.92) (McHugh, 2012).

### 2.3 Appraisal of evidence

Included papers were appraised based on rigor (e.g., whether the methods used to generate data is credible and trustworthy) and relevance (e.g., whether it can contribute to theory building) (Wong et al., 2013). In line with a realist philosophy, articles were not excluded or graded based on hierarchical assessment of study quality (Pawson et al., 2005). Assessment of relevance and rigor was conducted by review team members through discussion and based on sample size, data collection, data analysis, and conclusions. We followed the appraisal approach of Harden and colleagues where each article was assessed for relevance (fit within scope of review) and rigor (if conclusions aligned with research design) (Harden et al., 2015). For each item, we determined whether the article met the criteria or not (1 = yes, 0 = no). Only articles with a score of two were included in the synthesis.

### 2.4 Data extraction and synthesis

The data extraction form was developed with input from the research team and advisory group. Data extraction was first completed by one research assistant and confirmed for accuracy by a second (see supplementary file 2 for full data extraction).

To address the first research question, findings were organized to separate successful (e.g., resulting in positive improvements in physical activity behaviour or positive changes in health outcomes) and unsuccessful interventions (e.g., no change or statistically insignificant change) as reported in included papers. Using a narrative description, we summarized the intervention characteristics and feasibility of successful interventions to describe what works to promote physical activity in rural health and social care settings.

**Fig. 1:**
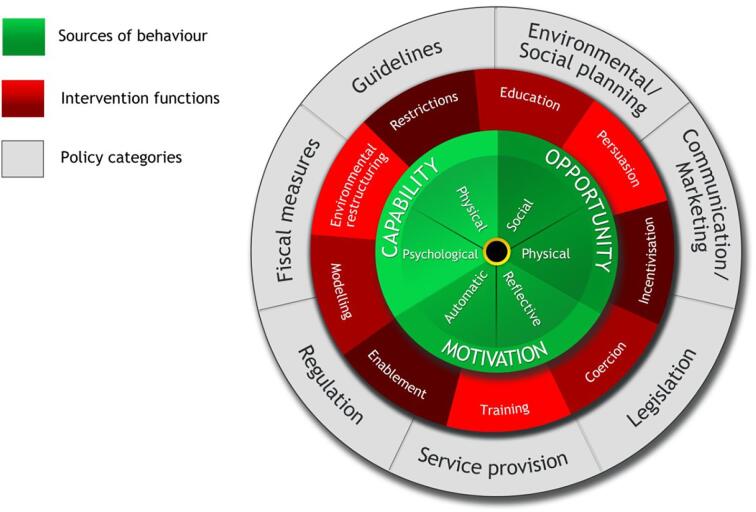
The Behaviour Change Wheel (From Michie et al., 2011, Michie et al., 2014).

To address the second research question, intervention mechanisms were identified by mapping intervention activities to the Behaviour Change Wheel (e.g., COM-B; Fig. 1). The Behaviour Change Wheel recognizes behaviour is influenced by a variety of factors and modification of the three constructs, capability (C), opportunity (O), and motivation (M; COM-B), can result in behaviour change (Michie et al., 2011, Michie et al., 2014). This model was chosen as it incorporates context naturally through internal and external factors influencing behaviour (Michie et al., 2011, Michie et al., 2014) and has been previously used to characterize intervention mechanisms in knowledge syntheses (Minian et al., 2020). To identify intervention context, we considered the social and cultural circumstances of intervention delivery (e.g., who delivered the intervention, community characteristics, and health care setting).

## 3 Results

**Fig. 2:**
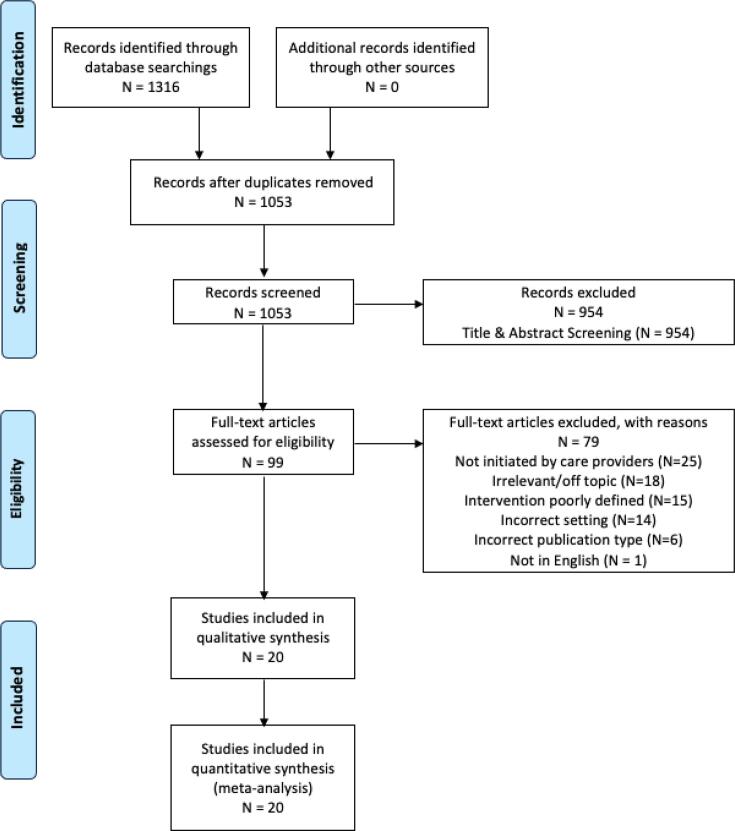
PRISMA Flow Diagram.

Our literature search yielded 1316 articles before the removal of duplicates. Following two levels of screening and targeted hand searches, our final sample consisted of 20 articles (see Fig. 2 for the PRISMA flow diagram). No articles were excluded based on assessment of rigor or study quality.

### 3.1 Study characteristics

Over half of the articles in our sample originated from the United States (n = 11) (Batsis et al., 2020, Batsis et al., 2021, Currie et al., 2018, Greaney et al., 2017, Melton et al., 2016, Peterson and Cheng, 2013, Reed et al., 2018, Reed et al., 2020, Sherman et al., 2007, Batsis et al., 2021, Robles et al., 2014), followed by Australia (n = 3) (Paul et al., 2019, Eakin et al., 2012, Sangster et al., 2016), Canada (n = 2) (Miedema et al., 2015, Davis et al., 2016), and the United Kingdom (n = 2; Table 3) (Connelly et al., 2017, Lee et al., 2007). Most studies were longitudinal or prospective cohort studies (n = 8) (Batsis et al., 2020, Batsis et al., 2021, Davis et al., 2016, Peterson and Cheng, 2013, Reed et al., 2020, Sherman et al., 2007, Batsis et al., 2021, Robles et al., 2014) or randomized control trials (n = 7) (Reed et al., 2018, Connelly et al., 2017, Greaney et al., 2017, Currie et al., 2018, Eakin et al., 2012, Lee et al., 2007, Sangster et al., 2016). Populations of interest included older adults (n = 7) (Batsis et al., 2020, Batsis et al., 2021, Davis et al., 2016, Hsu et al., 2018, Lee et al., 2007, Tarazona-Santabalbina et al., 2016, Batsis et al., 2021), individuals with chronic conditions and/or risk factors (n = 5) (Sangster et al., 2016, Miedema et al., 2015, Connelly et al., 2017, Paul et al., 2019, Eakin et al., 2012), women (n = 4) (Melton et al., 2016, Greaney et al., 2017, Sherman et al., 2007, Peterson and Cheng, 2013), and children/youth (n = 2) (Robles et al., 2014, Currie et al., 2018). The most common type of health care providers involved in intervention delivery were nurses or nurse practitioners (n = 8) (Connelly et al., 2017, Tarazona-Santabalbina et al., 2016, Sherman et al., 2007, Lee et al., 2007, Melton et al., 2016, Peterson and Cheng, 2013, Reed et al., 2020, Reed et al., 2018), physical therapists (n = 7) (Batsis et al., 2020, Batsis et al., 2021, Davis et al., 2016, Paul et al., 2019, Reed et al., 2018, Tarazona-Santabalbina et al., 2016, Batsis et al., 2021), registered dietitians (n = 5) (Batsis et al., 2020, Batsis et al., 2021, Greaney et al., 2017, Miedema et al., 2015, Batsis et al., 2021), physicians (n = 4) (Hsu et al., 2018, Miedema et al., 2015, Melton et al., 2016, Currie et al., 2018), and exercise physiologists (n = 3) (Paul et al., 2019, Miedema et al., 2015, Eakin et al., 2012). Two included studies reported on the same intervention delivered in a rural primary care setting, describing the effectiveness (Reed et al., 2018) and feasibility/implementation (Reed et al., 2020). We included three pilot or feasibility studies of different lengths and sample sizes based on a similar weight loss intervention (Batsis et al., 2020, Batsis et al., 2021, Batsis et al., 2021).

**Table 3:** Characteristics of included studies.

Characteristic	Number of included papers (n = 20)
**Year of publication**	
2005–2010	2
2011–2015	4
2016–2020	12
2021	2
**Country of origin**	
United States	11
Australia	3
Canada	2
United Kingdom	2
Other	2
**Study design**	
Pre/Post or prospective cohort	8
Randomized control trial	7
Quasi-experimental	4
Feasibility	1
**Study population**	
Older adults	7
Existing conditions/risk factors	5
Women	4
Children and adolescents	2
Adults	2
**Health care provider**	
Nurses/nurse practitioners	8
Physical therapists	7
Registered dietitians/ nutritionists	6
Physicians/ physician assistants	5
Exercise physiologists	3
Health coaches	2
Other health professionals and specialists	5

Note: some columns add to more than the number of included papers due to multiple health care providers or population groups included in a single study.

### 3.2 Rural community context

Ten articles (Hsu et al., 2018, Paul et al., 2019, Connelly et al., 2017, Greaney et al., 2017, Miedema et al., 2015, Melton et al., 2016, Robles et al., 2014, Sangster et al., 2016, Batsis et al., 2021, Currie et al., 2018) provided no definition or measure of rurality but were included as they self-identified their study as taking place in a rural setting. Seven articles defined the communities as rural by stating the location’s population (range: 5,495 – 20,613 people) (Davis et al., 2016, Lee et al., 2007, Peterson and Cheng, 2013, Reed et al., 2020, Sherman et al., 2007, Tarazona-Santabalbina et al., 2016, Batsis et al., 2021), and three provided a specific definition of rurality based on national health policies (Batsis et al., 2020, Reed et al., 2018, Eakin et al., 2012).

### 3.3 Outcomes: physical activity and health status

Nine articles recorded improvements in physical activity behaviour (Reed et al., 2018, Hsu et al., 2018, Paul et al., 2019, Tarazona-Santabalbina et al., 2016, Sherman et al., 2007, Melton et al., 2016, Robles et al., 2014, Sangster et al., 2016, Currie et al., 2018) and five of these studies reported a statistically significant increase (Melton et al., 2016, Paul et al., 2019, Sangster et al., 2016, Sherman et al., 2007, Robles et al., 2014) (Table 4). The five studies with statistically significant increases in physical activity behaviour included a hybrid health coaching and weekly exercise tracking intervention (Paul et al., 2019), a walking intervention (Sherman et al., 2007), a health education campaign at an obstetric and gynecology clinic (Melton et al., 2016), a pedometer-based telephone coaching intervention (Sangster et al., 2016), and a diet and supervised exercise intervention (Robles et al., 2014).

**Table 4:** Characteristics of interventions with statistically significant outcomes related to physical activity.

Article Author(s)	Study Location	Population	Provider Involved	Intervention	Intervention activities	User Experience
**Changes in: Physical Activity Levels**						
(Sherman et al., 2007)	USA	Women	Nurse practitioner,Nurse	Walking promotion intervention	Motivational interviewing & counselling; Fitbit, pedometer, activity tracker; Take home materials (DVD, videos, photos, handouts);	80% Retention
(Sangster et al., 2016)	Australia	Referred for cardiac rehabilitation	Health Coaches	Physical activity promotion intervention	Fitbit, pedometer, activity tracker; Take home materials (DVD, videos, photos, handouts); Check-ins & reminders (Phone, text, email, or app)	91% Retention for rural group, 93% for urban and semi-rural
**Changes in: Physical Activity Levels, Fitness/Physical Functionality**						
(Paul et al., 2019)	Australia	Chronic disease	Physical therapist, Exercise Physiologist, Students	Exercise and healthy lifestyle program	Exercise plan or prescription, Goal setting, Check-ins & reminders (Phone, text, email, or app)	63% Retention, 59% of participants attained at least one of their health-related goals
**Changes in: Physical Activity Levels; Weight, Waist Circumference, BMI**						
(Robles et al., 2014)	USA	Children	Pharmacists	Community and park based physical activity promotion and nutrition education intervention	Exercise plan or prescription, Diet plans & nutritional interventions, Group exercise activities	94% Retention
**Changes in: Fitness/Physical Functionality**						
(Tarazona-Santabalbina et al., 2016)	Spain	Older people	Physical therapist and Nurse	Functional exercise Intervention	Exercise plan or prescription, Aerobic exercise, Strength training	Not reported
(Davis et al., 2016)	Canada	Older people	Physical therapist	Functional exercise intervention to prevent falls	Exercise plan or prescription, Take home materials (DVD, videos, photos, handouts), Aerobic exercise, Strength training	Compliance data collected on 72% of participants, 87% compliance for exercise program, 166% compliance with walking
(Lee et al., 2007)	United Kingdom	Older people	Nurse	Community based walking intervention	Fitbit, pedometer, activity tracker; Exercise diary or tracker; Weekly lectures or education sessions	83% Retention, higher adherence to walking in intervention group
**Changes in: Fitness/Physical Functionality; Weight, Waist Circumference, BMI**						
(Batsis et al., 2020)	USA	Older people	Physical therapist and dietician	Exercise and diet weight-loss intervention	Motivational interviewing & counselling, Exercise plan or prescription, Goal setting, Diet plans & nutritional interventions, Exercise diary or tracker, behavioural and psychological support	100% Retention, 88–89% Adherence
(Batsis et al., 2021)	USA	Older people	Physical therapist, Dietician	Exercise and diet intervention	Fitbit, pedometer, activity tracker; Exercise plan or prescription; Diet plans & nutritional interventions; Exercise diary or tracker; Aerobic exercise; Strength training	84.8% Retention; 91.9–93.8% Adherence to nutrition/behavioural sessions, physical therapy sessions, and Fitbit use
(Batsis et al., 2021)	USA	Older people	Physical therapist, Dietician	Exercise and diet weight management program	Motivational interviewing & counselling; Fitbit, pedometer, activity tracker; Take home materials (DVD, videos, photos, handouts); Exercise plan or prescription; Goal setting; Diet plans & nutritional interventions; Group exercise activities	83% Retention; 77–78.2% Adherence to physical therapy visits, 84–90% to Dietician visits, 81.7% average Fitbit use
**Changes in: Physical activity, health Knowledge**						
(Melton et al., 2016)	USA	Prenatal and patients of reproductive age	Physician, Nurse, Physician Assistant, Ultrasound Tech	Information campaign	Passive information campaign	Not reported

Note: Outcomes included physical activity levels (i.e., steps per day, hours per week); fitness/physical functionality changes (i.e., 6 min walk test, Max Gait Speed, Mean Grip Strength, Times Sit To Stand); weight, waist circumference, and BMI; nutrition (i.e., glycosylated hemoglobin, reported diet); health knowledge; and mental health (i.e. self-efficacy, coping strategies).

Eight articles reported statistically significant improvements in fitness or physical function (Batsis et al., 2020, Batsis et al., 2021, Davis et al., 2016, Lee et al., 2007, Paul et al., 2019, Sangster et al., 2016, Tarazona-Santabalbina et al., 2016, Batsis et al., 2021). Improvements were measured using the 6-minute walk test (n = 3) (Batsis et al., 2020) and timed in sit to stand (n = 3) (Batsis et al., 2020, Batsis et al., 2021, Batsis et al., 2021). Improvements were noted in max gait speed (n = 2) (Batsis et al., 2020, Batsis et al., 2021), mean grip strength (n = 1) (Batsis et al., 2020), reduced fall risk (n = 1) (Davis et al., 2016), reduced systolic blood pressure (n = 1) (Lee et al., 2007), reduced/reversed frailty (n = 1) (Tarazona-Santabalbina et al., 2016), or by fewer hospital visits (n = 1) (Paul et al., 2019). All articles used p values to indicate statistical significance and one article reported practically and clinically important effect sizes (Sangster et al., 2016).

### 3.4 Outcomes: intervention evaluation

Nine articles reported a high retention rate (e.g., number of participants lost to follow-up) of 75–100% with interventions including both nutrition and physical therapy/exercise sessions (n = 4) (Batsis et al., 2020, Batsis et al., 2021, Batsis et al., 2021, Robles et al., 2014), web/telephone-based interventions (n = 3) (Connelly et al., 2017, Eakin et al., 2012, Sangster et al., 2016) or walking interventions (n = 2) (Sherman et al., 2007, Lee et al., 2007). Three articles had moderate retention (50–74%) (Paul et al., 2019, Greaney et al., 2017, Currie et al., 2018), one had low retention (25–49%) (Miedema et al., 2015).

Twelve articles reported intervention adherence (e.g., percent of sessions attended), with six reporting high adherence rates between 75 and 100% (Batsis et al., 2020, Batsis et al., 2021, Eakin et al., 2012, Lee et al., 2007, Batsis et al., 2021(Davis et al., 2016)). The articles reporting high adherence involved both nutrition and physical therapy/exercise sessions (n = 3) (Batsis et al., 2020, Batsis et al., 2021, Batsis et al., 2021), a walking intervention (Lee et al., 2007), and a telephone-based intervention (Eakin et al., 2012). Three articles reported relatively low adherence (between 40 and 75%) (Reed et al., 2018, Greaney et al., 2017, Miedema et al., 2015). Three articles described adherence based on general trends/use of intervention components (e.g., increase in hours of planned exercise, drops in log-ins for the website, and number of times intervention calls were completed) (Connelly et al., 2017, Peterson and Cheng, 2013, Currie et al., 2018).

Fourteen articles (Batsis et al., 2020, Batsis et al., 2021, Connelly et al., 2017, Currie et al., 2018, Eakin et al., 2012, Greaney et al., 2017, Lee et al., 2007, Miedema et al., 2015, Paul et al., 2019, Peterson and Cheng, 2013, Reed et al., 2018, Reed et al., 2020, Sherman et al., 2007, Batsis et al., 2021) reported an evaluation of user experience or feasibility by measuring participant satisfaction with the intervention or technological components. Common issues discussed were financial barriers of the intervention (for the provider and participant) (Greaney et al., 2017, Peterson and Cheng, 2013), concerns about functionality of the technology/tools used (Hsu et al., 2018, Reed et al., 2018, Eakin et al., 2012, Reed et al., 2020, Batsis et al., 2021), and the success of participants meeting intervention goals.

### 3.5 Intervention mechanisms

Intervention activities were identified (Table 5) and mapped to the COM-B model (Table 6). Of the interventions reporting statistically significant improvements in physical activity or health outcomes, common intervention activities included the use of a wearable tracking device (e.g., FitBit, pedometer) (Lee et al., 2007, Sangster et al., 2016, Sherman et al., 2007); check-ins from trusted sources (e.g. smartphone applications, websites, virtual calls, motivational texts, and telephone conversations) (Paul et al., 2019, Sangster et al., 2016); and personalized exercise prescriptions (Batsis et al., 2020, Batsis et al., 2021, Davis et al., 2016, Paul et al., 2019, Tarazona-Santabalbina et al., 2016, Batsis et al., 2021, Robles et al., 2014) Motivational interviewing and counselling (Batsis et al., 2020, Batsis et al., 2021, Sherman et al., 2007) and take home materials and resources (e.g. exercise DVDs, resistance bands, photo guides, and handouts) (Davis et al., 2016, Sangster et al., 2016, Sherman et al., 2007), were each used in three successful interventions. Three successful interventions involved exercise diaries and journals (Batsis et al., 2020, Lee et al., 2007, Batsis et al., 2021).

**Table 5:** Intervention strategies of included studies.

	(Batsis et al., 2020)	(Reed et al., 2018)	(Hsu et al., 2018)	(Paul et al., 2019)	(Connelly et al., 2017)	(Greaney et al., 2017)	(Tarazona-Santabalbina et al., 2016)	(Miedema et al., 2015)	(Davis et al., 2016)	(Eakin et al., 2012)	(Sherman et al., 2007)	(Lee et al., 2007)	(Melton et al., 2016)	(Peterson and Cheng, 2013)	(Robles et al., 2014)	(Reed et al., 2020)	(Sangster et al., 2016)	(Batsis et al., 2021)	(Batsis et al., 2021)	(Currie et al., 2018)	Successful interventions	Totals
Motivational interviewing & counselling	**✓**	✓				✓				✓	**✓**			✓					**✓**	✓	3	8
Fitbit, pedometer, activity tracker		✓				✓					**✓**	**✓**					**✓**	**✓**	**✓**	✓	5	8
Exercise plan or prescription	**✓**			**✓**			**✓**		**✓**						**✓**			**✓**	**✓**		7	7
Goal setting	**✓**			**✓**	✓	✓		✓											**✓**	✓	3	7
Take home materials (DVD, videos, photos, handouts)					✓				**✓**	✓	**✓**			✓			**✓**			✓	3	7
Check-ins & reminders (Phone, text, email, or app)		✓		**✓**		✓				✓							**✓**			✓	2	6
Diet plans & nutritional interventions	**✓**							✓							**✓**			**✓**	**✓**		4	5
Exercise diary or tracker	**✓**				✓					**✓**		**✓**						**✓**			3	5
Group exercise activities								✓						✓	**✓**				**✓**		2	4
Behavioural and psychological support	**✓**					✓		✓													1	3
Aerobic exercise							**✓**		**✓**									**✓**			3	3
Strength training							**✓**		**✓**									**✓**			3	3
Weekly lectures or education sessions			✓									**✓**									1	2
Planning models (5A)		✓																			0	1
Quizzes			✓																		0	1
Interactive web portal					✓																0	1
Maps with local PA destinations					✓																0	1
Gym membership						✓															0	1
Resistance bands			✓																		0	1
Passive information campaign													**✓**								1	1
Participation awards and celebration															**✓**						1	1
Qualitative evaluation of experience																✓					0	1

Legend: ✓ indicates mechanical used, bolding indicates positive effect of intervention strategy/component on physical activity measures (successful interventions).

**Table 6:** Intervention mechanisms mapped to Behaviour Change Wheel (COM-B).

Study authors	Capability – physical	Capability – psychological	Opportunity – physical	Opportunity – social	Motivation – automatic	Motivation – reflective
(Batsis et al., 2020)		**✓**	**✓**			**✓**
(Reed et al., 2018)			✓			✓
(Hsu et al., 2018)	✓	✓	✓			
(Paul et al., 2019)			**✓**			**✓**
(Connelly et al., 2017)	✓	✓	✓		✓	✓
(Greaney et al., 2017)	✓		✓			✓
(Tarazona-Santabalbina et al., 2016)	**✓**					
(Miedema et al., 2015)		✓		✓		
(Davis et al., 2016)	**✓**					
(Eakin et al., 2012)			✓			✓
(Sherman et al., 2007)	**✓**		**✓**			**✓**
(Lee et al., 2007)		**✓**	**✓**			
(Melton et al., 2016)		**✓**	**✓**			
(Peterson and Cheng, 2013)	✓	✓	✓			✓
(Robles et al., 2014)	**✓**	**✓**		**✓**	**✓**	**✓**
(Reed et al., 2020)			✓			✓
(Sangster et al., 2016)		**✓**	**✓**			**✓**
(Batsis et al., 2021)	**✓**		**✓**			
(Batsis et al., 2021)	**✓**	**✓**	**✓**			**✓**
(Currie et al., 2018)	✓		✓			✓

Legend: ✓indicates component present in intervention, boldiing indicates positive changes in physical activity related measures.

Based on the COM-B model, social opportunity was successful in initiating positive change in physical activity behaviour in one of the two studies where the mechanism was used (Robles et al., 2014). Similarly, automatic motivation was successful in one of two instances (Robles et al., 2014). Reflective motivation mechanisms were identified in 13 studies, and were successful across six interventions (Batsis et al., 2020, Batsis et al., 2021, Paul et al., 2019, Sangster et al., 2016, Sherman et al., 2007, Robles et al., 2014). Physical opportunity was the most implemented mechanism and was successful in eight of 16 studies (Batsis et al., 2020, Batsis et al., 2021, Lee et al., 2007, Melton et al., 2016, Paul et al., 2019, Sangster et al., 2016, Sherman et al., 2007, Batsis et al., 2021). Psychological capability mechanisms were successful in six of 10 interventions (Batsis et al., 2020, Batsis et al., 2021, Lee et al., 2007, Melton et al., 2016, Sangster et al., 2016, Robles et al., 2014). Finally, physical capability mechanisms were used in 11 studies, and were successful in six (Batsis et al., 2021, Davis et al., 2016, Sherman et al., 2007, Tarazona-Santabalbina et al., 2016, Batsis et al., 2021, Robles et al., 2014).

## 4 Discussion

The aim of this project was to increase understanding of which interventions are effective in promoting physical activity in rural health and social care settings and to identify mechanisms and contextual factors impacting intervention delivery. Our findings identify motivational interviewing, physical activity counselling, exercise plans or prescription, and the use of wearable activity trackers as the most common intervention strategies. The most successful intervention strategies (e.g., those positively influencing physical activity behaviour) included wearable activity trackers, and check-ins or reminders from trusted sources. Interventions with mechanisms involving physical opportunity, automatic motivation, and psychological capability were more likely to be successful than other factors of the COM-B model

### 4.1 What works to promote physical activity in rural health care settings?

Successful intervention strategies included a method for tracking progress, providing counselling and/or exercise prescriptions, and incorporating follow-up reminders to prompt behaviour change. These intervention strategies or activities align with intervention targets related to capability, motivation, and opportunity,respectively. Previous work has identified psychological capability and reflective motivation as predictive of moderate-to-vigorous intensity physical activity in the general adult population (Howlett et al., 2019). Among included studies, the COM-B components most associated with successful interventions were similar, aligning with psychological capability, automatic motivation, and physical opportunity. These findings suggest focusing on intrinsic behaviour processes (e.g., motivation, self-efficacy) alongside addressing external environmental influences (e.g., opportunities to be active) may be necessary to change physical activity behaviour in rural communities. Social opportunity was also identified as an important aspect of the COM-B model indicating the social environment may impact decision-making and physical activity behaviour for rural residents. The relatively small number of studies included in our review limits our ability to draw conclusions for future intervention design or pool study findings.

Like other reviews on physical activity interventions delivered in rural communities (Pelletier et al., 2020), included studies reported variable effectiveness in improving physical activity behaviour with only five studies reporting a significant increase after periods of 12 weeks to six months (Sherman et al., 2007, Robles et al., 2014, Sangster et al., 2016, Melton et al., 2016, Paul et al., 2019). While a lack of intervention effectiveness may reflect complexity and challenges associated with increasing physical activity behaviour, it may also reflect poor adaptation or intervention development for a rural context (e.g., poor implementation). Although community engagement was not fully described in the majority of included studies, four studies described efforts to incorporate local perspectives into intervention design including focus groups with community members (Connelly et al., 2017), meetings with clinic staff (Melton et al., 2016), or partnering with local knowledge users (Robles et al., 2014, Reed et al., 2020). The resources, population, and environment differ between communities and can impact intervention implementation in rural areas (Pelletier et al., 2020). A community-centered approach may be essential for intervention success, with communities themselves identifying their priorities, barriers or challenges, and consequently feasibility of a given intervention. Future studies should include appropriate implementation related outcomes (e.g., dose, fidelity) and describe adapation processes to determine characteristics of successful intervention delivery in rural communities. Of the included studies describing intervention feasibility, participants seemed to enjoy tracking and goal setting aspects of intervention delivery (thus improving study retention). Factors such as difficulty or barriers interacting with technology components and changes in personal health or family circumstances contributed to low intervention adherence.

### 4.2 Defining rurality and intervention context

We note some contextual factors of intervention delivery, such as the broad range of health care providers involved, but given the relatively small sample of included papers and the broad range of outcomes and intervention strategies, we are unable to draw conclusions linking context-mechanism-outcome. An important finding from our review was the conceptualization of physical activity as a clinical intervention rather than a preventive population health strategy. Few articles aimed to solely increase physical activity; most articles aimed to increase physical activity in the context of another aim (e.g., weight loss, obesity prevention), to treat or manage disease symptomology, or to reduce frailty. The clinical application of physical activity (e.g., exercise is medicine) offers an important indication of how physical activity is viewed and promoted in health care settings. While the benefits of physical activity for the general population are thoroughly evidenced in the literature (Chastin et al., 2021, Lee et al., 2012), physical activity interventions in rural communities are often targeted at specific demographics and outcomes, versus general community health and wellbeing. For rural populations who tend to participate less in physical activity (Pelletier et al., 2021), a multi-system, whole of community approach may be needed to address deeply rooted health and physical activity inequities, particularly given the inequitable distribution of health care providers and exercise professionals between urban and rural communities.

Among the included studies, physical therapists were one of the health care providers most likely to be involved in intervention delivery. All interventions involving physical therapsists (e.g., leading group-based exercise, prescription of exercise, telephone coaching), reported positive improvements in health or physical activity outcomes. A possible explanation for the success observed in physical therapist-led interventions could be due to patient/participant perception of physical therapists as experts in physical activity. This success may also be attributed to the length of time spent with patients, and increased communication and connection between physical therapists and patients/participants compared to other providers, particularly in rural areas where people are less likely to have regular contact with a family physician (Shah et al., 2020). While physicians may have limited time to discuss physical activity, physical therapists spend more time and focus on exercise and physical activity as part of their role (Lowe et al., 2018). The role of physical therapists as physical activity advocates may be amplified in the context of the included interventions, as physical therapists often led exercise classes, and established personalized exercise plans for participants, thus forming individualized connections and familiarity which may have encouraged open communication, personalization, and discussion of physical activity.

The lack of a consistent definition of rurality was expected and has been noted in previous reviews (Pelletier et al., 2020, Bhuiyan et al., 2019). Inconsistent definitions of rurality reflect the diversity of communities captured in our sample and the difficulties in defining a rural context. As noted by Greenhalgh & Manzano (Greenhalgh and Manzano, 2021), there is no universal approach to describing and defining context. Often, intervention studies or randomized controlled trials describe context as a set of items to document in order to minimize or control rather than something shaping the complex system where an intervention is delivered (Pfadenhauer et al., 2017). A realism lens considers the broad scope of context, including identifying ‘what works, and in which circumstances does it work’ (Pawson et al., 2005). While many included studies described their work as being conducted in a rural setting, the poorly defined context of the rural setting (often based on a quantitative characteristic such as distance from an urban centre or population size) creates challenges for synthesis and understanding how rurality impacts intervention outcomes. Rural health care systems are characterized by relationships, autonomy, and sharing of resources (Nelson et al., 2021, Gessert et al., 2015, Hart et al., 2005, Johnston et al., 2021). While we anticipate these factors shaped intervention mechanisms and success, the poor description of adaptation to rural contexts limits our ability to connect context with intervention outcomes.

### 4.3 Limitations

Due to the expansive and varied terminology used to define rurality and remoteness, it is possible some interventions were missed in our search. While hand searching was performed to mitigate challenges in categorizing rurality, we balanced the need for comprehensiveness and speed, thus, we do not consider our review to be exhaustive. We did not included a specific systematic grey literature search, which might have captured additional interventions and outcomes delivered by government and non-profit sectors not published in traditional academic literature. Our identification of studies as being successful or not was based on the statistical tests and reporting of the original papers and we are not able to comment on effect sizes and/or clinical significance of outcome measures.

Using the COM-B model enabled the deconstruction of general intervention mechanisms used to elicit physical activity behaviour change in rural populations. The COM-B variables can be mapped to the Behaviour Change Wheel for specific recommendations for intervention design for future research. Many of the included studies provided a limited description of the intervention and few used a behaviour change theory to guide intervention development, imposing challenges in assigning or isolating specific COM-B mechanisms. Several studies included more than one COM-B construct, and we are unable to determine the degree of overlap. Due to a mediating effect of COM-B constructs on physical activity behaviour (Howlett et al., 2019), the overlap of mechanisms further limits understanding of the relationship between intervention mechanisms and outcomes.

## 5 Conclusion

Among the identified studies focusing on physical activity promotion in rural health care settings, there is a broad classification of rurality and varied intervention mechanisms. While some studies reported an increase in physical activity behaviour, the majority of included studies focused on noncommunicable disease risk reduction rather than physical activity promotion as a strategy to improve overall health and wellbeing. The addition of wearable activity trackers and support from health care providers may be an important element of intervention success. Future work should explore how the rural context impacts intervention delivery and success, and identify strategies to work with community members to deliver appropriate interventions reflective of rural active living environments.

## Funding

This project was funded by a Team Building Award from the Rural Health Services Research Network of British Columbia and further supported by the Canadian Institutes of Health Research (Grant number: UD1-170258).

## Declaration of Competing Interest

The authors declare that they have no known competing financial interests or personal relationships that could have appeared to influence the work reported in this paper.
