# Supplementary material for: Physical activity promotion in rural health care settings: A rapid realist review
Source: Prev Med Rep. 2022 Jul 9;29:101905. doi: 10.1016/j.pmedr.2022.101905 (PMC9307466; doi:10.1016/j.pmedr.2022.101905)
Supplement: Supplementary Data 1 [file mmc1.docx]

**Additional File: Medline EBSCO Search Strategy**
Limiters: Full text; English language; Human.
Date: May 4, 2021

| **#** | **Searches** | **Results** |
| --- | --- | --- |
| 1 | (MH "Exercise") OR TI "Exercise" OR AB "Exercise" OR TI "Exercises" OR AB "Exercises" OR TI “Physical Activity” OR AB “Physical Activity” OR TI “Physical Activities” OR AB “Physical Activities” OR TI “Physical Exercise” OR AB “Physical Exercise” OR TI “Physical Exercises” OR AB “Physical Exercises” OR TI “Acute Exercise” OR AB “Acute Exercise” OR TI “Acute Exercises” OR AB “Acute Exercises” OR TI “Exercise Training” OR AB “Exercise Training” OR TI “Exercise Trainings” OR AB “Exercise Trainings” OR TI “Ambulation” OR AB “Ambulation” OR TI “Human Physical Conditioning” OR AB “Human Physical Conditioning” OR TI “Human Physical Training” OR AB “Human Physical Training” OR Active living OR lifestyle OR movement OR recreation OR fitness | 415212 |
| 2 | TI (healthcare) OR (health care) N2 (provider) | 94528 |
| 3 | Rural OR TIAB remote OR TIAB northern OR TIAB Indigenous OR TIAB Aboriginal OR TX non-urban OR TIAB ((rural) N2 (community)) | 202085 |
| 4 | S2 and S3 | 3806 |
| 5 | S1 and S4 | 77 |
| 6 | ((prescription N2 (exercise) OR (program)) OR TX (exercise) N2 (program) OR (intervention) OR (promotion) OR (counselling) OR TX (behaviour) OR (behavior) N2 (change) OR TX feasibility | 2901662 |
| 7 | S5 and S6 | 44 |
| 8 | S1 and S2 and S3 and S6 | 44 |
| 9 | ((MH "Randomized Controlled Trials") OR TI "Randomized Controlled Trials" OR AB "Randomized Controlled Trials" OR (MH "Clinical Trials") OR TI "Clinical Trials" OR AB "Clinical Trials" OR (MH "Health Promotion") OR TI "Health Promotion" OR AB "Health Promotion" OR (MH "Weight Reduction Programs") OR TI "Weight Reduction Programs" OR AB "Weight Reduction Programs" OR (MH "Experimental Studies") OR TI "Experimental Studies" OR AB "Experimental Studies" OR (MH "Intervention Trials") OR TI "Intervention Trials" OR AB "Intervention Trials" OR TI "Intervention" OR AB "Intervention" OR TI "Wellness Program" OR AB "Wellness Program" OR TI "Obesity Management" OR AB "Obesity Management”) OR TI ((prescription) N2 (exercise OR program*)) OR TX (exercise) N2 (program*) OR (intervention*) OR (promotion*) OR (counselling) OR TX (behaviour OR behavior) N2 (change) OR TX feasibility OR TI model* OR TI strateg* OR TIAB community-based program | 1006355 |
| 10 | S8 and S9 | 23 |
| 11 | TI (healthcare) OR (health care) N2 (provider*) OR TIAB (interdisciplinary) OR (interprofessional) N2 (team*) OR TIAB physician* OR TIAB nurse* OR TIAB (nurse) N2 (practitioner*) OR TIAB physiotherap* OR TIAB mental health OR TIAB counsellor* OR TIAB dietitian* OR TIAB (occupational OR exercise) OR (recreation*) N2 (therapist*) OR TIAB pharmac* OR TIAB kinesiolog* OR (physiolog*) OR TIAB (social) N2 (worker*) OR TIAB (care) N2 (aide*) OR TIAB (physiotherapist*) OR (occupational) N2 (assistant*) OR (occupational) N2 (therapist*) OR TIAB community health worker* OR TIAB community health care worker* OR TIAB community-based program | 4979102 |
| 12 | S1 and S3 and S6 and S9 and S11 | 350 |
